# Hémodialyse chronique et dépression au Centre Hospitalier Universitaire Sylvanus Olympio de Lomé (Togo)

**DOI:** 10.11604/pamj.2016.25.26.9883

**Published:** 2016-09-27

**Authors:** Mawufemo Yawovi Tsevi, Saliou Salifou, Akomola Kossi Sabi, Befa Noto-Kadou-Kaza, Eyram Yoan Amekoudi, Simliwa Kolou Dassa

**Affiliations:** 1 Service de Néphrologie et d’Hémodialyse, CHU Sylvanus Olympio, Lomé-Togo; 2 Clinique Universitaire de Psychiatrie et de Psychologie Médicale du CHU Sylvanus Olympio, Lomé, Togo

**Keywords:** Dépression, Insuffisance rénale chronique, Hémodialyse, Depression, chronic renal failure, hemodialysis

## Abstract

**Introduction:**

Estimer la prévalence de la dépression et rechercher les facteurs associés chez les patients insuffisants rénaux chroniques en hémodialyse.

**Méthodes:**

Étude transversale descriptive allant du 1er Janvier 2014 au 31 Décembre 2014 à l’unité d’hémodialyse du service de Néphrologie du CHU Sylvanus Olympio de Lomé (Togo). L’échelle d’autoévaluation de la dépression de Beck dans sa version simplifiée a été notre outil d’évaluation.

**Résultats:**

Durant la période d’étude, 88 patients ont été recrutés dont 61,4% d’hommes soit un sex-ratio de 1,6. La moyenne d’âge a été de 38,80 ± 13,24 ans avec des extrêmes de 12 et 66 ans. La majorité des patients (90,9%) étaient des travailleurs. L’hypertension artérielle a été la comorbidité somatique la plus retrouvée (45,4%). Quarante-six patients (52,3%) avaient une durée en hémodialyse comprise entre 1 et 4 ans. La dépression touchait 68,2% des patients; 47,7% des patients déprimés avaient une dépression sévère. La survenue de la dépression était significativement liée à la durée en hémodialyse (p= 0,008).

**Conclusion:**

La prise en charge du patient hémodialysé chronique doit être pluridisciplinaire incluant le néphrologue et le psychiatre.

## Abstract (en)

**Introduction:**

The aim of our study was to estimate the prevalence of depression and to investigate its associated risk factors in patients with chronic renal failure on hemodialysis.

**Methods:**

We conducted a descriptive cross-sectional study in the hemodialysis unit of the Division of Nephrology at the University Hospital of Sylvanus Olympio Lomé (Togo) from 1 January 2014 to 31 December 2014. Self-evaluation Scale as calibrated using the Beck Depression Inventory in his simplified version was our screening tool.

**Results:**

During the study period, 88 patients were enrolled of whom 61.4% were men with a sex ratio of 1.6. The average age was 38.80 ± 13.24 years ranging from 12 to 66 years. The majority of patients (90.9%) were workers. Arterial hypertension was the most common somatic comorbidity (45.4%) recorded. Forty-six patients (52.3%) had hemodialysis duration between 1-4 years; 68.2% of patients had depression; 47.7% of depressed patients had severe depression. The occurrence of the depression is significantly related to the hemodialysis length (p= 0,008).

**Conclusion:**

The management of chronic hemodialysis patient should be multidisciplinary including nephrologist and psychiatrist.

## Introduction

La dépression est une affection courante dans le monde. Selon les estimations de l’OMS, 350 millions de personnes en souffrent et il existe une interdépendance entre la dépression et la santé physique [1]. L'évolution de la pathologie dépressive est parfois aggravée par l'existence de certaines maladies organiques. De même, les sujets présentant un état dépressif associé connaissent une évolution plus sévère de leur pathologie somatique, avec un risque de décès accru [2]. Pour les patients insuffisants rénaux au stade de la dialyse, la sévérité des symptômes dépressifs est associée à une mortalité plus élevée [3–5]. Au Togo, aucune étude sur la dépression chez les patients souffrant d’insuffisance rénale chronique en général et en hémodialyse en particulier n’a été réalisée. Cette étude avait pour objectif: d’évaluer la prévalence de la dépression et de rechercher les facteurs associés chez les patients insuffisants rénaux chroniques en hémodialyse.

## Méthodes

### Cadre et Méthode d’étude

#### Cadre d’étude

Notre étude a été réalisée dans l’unité d’hémodialyse du CHU Sylvanus Olympio de Lomé au Togo. Cette unité qui est l’unique centre publique de prise en charge en hémodialyse du pays, est gérée par un (01) médecin Néphrologue, 8 infirmiers et 3 techniciens de surface. Outre ces activités de soins, l’unité accomplit également des activités de formations et de recherches.

#### Méthode d’étude

**Type et période d’étude:** Il s’est agi d’une étude descriptive transversale allant du 1^er^ Janvier 2014 au 31 Décembre 2014 soit une période de 12 mois, chez des patients insuffisants rénaux chroniques pris en charge en hémodialyse périodique et qui avaient donné leur consentement libre et éclairé pour participer à l’étude.

**Population d’étude:** Elle est constituée à partir d’un échantillonnage exhaustif par choix raisonné des patients de l’unité d’hémodialyse du Centre Hospitalier et Universitaire Sylvanus Olympio de Lomé au Togo qui ont répondu aux critères suivants: **Critères d’inclusion:** Ont été inclus dans notre étude, les sujets des deux sexes, tout âge compris, pris en charge dans l’unité d’hémodialyse du CHU Sylvanus Olympio de Lomé au cours de la période d’étude et qui ont donné leur consentement libre et éclairé; **Critères de non inclusion:** N’ont pas été inclus dans cette étude, les patients qui ont refusé de participer à l’étude.

**Technique de collecte des données:** Les données ont été collectées à l’aide d’une fiche d’enquête préétablie comportant les principales variables suivantes: âge, sexe, profession, comorbidités somatiques, durée de l’hémodialyse. Nous avons recherché la dépression chez les patients grâce à l’échelle d’autoévaluation de la dépression de Beck dans sa version simplifiée à 13 items.

**Technique d’analyse des données:** La saisie et l’analyse statistique des données ont été faites au moyen du logiciel Statiscal Package for Social Science (SPSS) dans sa version 2.4. Une analyse univariée (seuil de significativité de 5%) a été réalisée à l’aide du test du chi2. Les tableaux et les figures ont été réalisés au moyen du logiciel EXCEL 2013.

## Résultats

### Résultats généraux

Durant la période d’étude 91 patients étaient pris en charge dans l’unité d’hémodialyse du CHU Sylvanus Olympio de Lomé. Quatre-vingt-huit patients ont répondu aux critères d’inclusion; 3 patients ont refusé de participer à l’étude. Parmi les 88 patients il y avait 54 hommes (61,4%) et 34 femmes (38,6%) ce qui correspondait à un sex-ratio à 1,6. La moyenne d’âge était de 38,80 ± 13,24 ans avec des extrêmes de 12 et 66 ans. La majorité des patients (90,9%) étaient des travailleurs. Tous les patients étaient admis dans l’unité d’hémodialyse au stade terminal de l’insuffisance rénale chronique (IRC). Quarante-six patients (52,3%) avaient une durée en hémodialyse comprise entre 1 et 4 ans.

### Comorbidités

**Figure 1:**
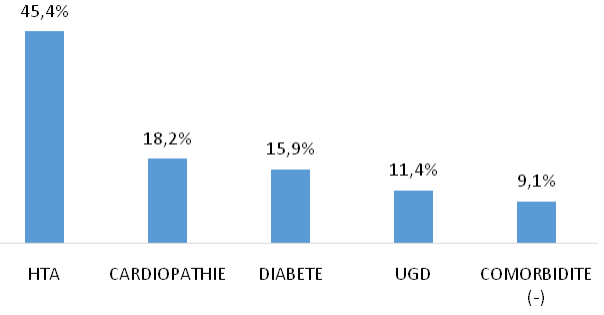
Répartition des patients en fonction de la comorbidité somatique

**Comorbidités somatiques:** l’hypertension artérielle a été la comorbidité la plus retrouvée (45,4%) comme le montre la Figure 1.

**Figure 2:**
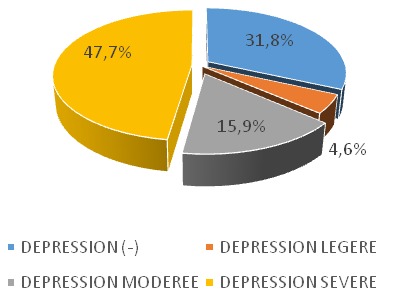
Répartition des patients en fonction du degré de sévérité de la dépression

**La dépression:** aucun patient n’avait un antécédent dépressif. La dépression était retrouvée chez 60 patients (68,2%) dont 38 hommes et 22 femmes. Aucun d’eux n’a bénéficié d’un traitement antidépresseur. Parmi les patients déprimés, 42 patients (47,7%) avaient une dépression sévère comme le montre la Figure 2. La survenue de la dépression était significativement liée à la durée en hémodialyse (p= 0,008). La survenue de la dépression n’était pas liée à l’existence de comorbidités somatiques (p=0,221) ni au statut professionnel (p=0,759) (Tableau 1).

**Tableau 1:** Lien entre la dépression, le sexe, l’âge, le statut professionnel, la durée en hémodialyse et les comorbidités somatiques

Caractéristiques des patients	Dépression					
	Oui		Non		Total	p-value
	Effectif	%	Effectif	%	Effectif	
**Sexe**						**0,349**
Masculin	34	63,0	20	37,0	54	
Féminin	26	76,5	8	23,5	34	
Total	60	68,2	28	31,8	88	
**Age**						**0,518**
Moins de 20 ans	0	0	2	100,0	2	
21-30 ans	20	71,4	8	28,6	28	
31-50 ans	24	70,6	10	29,4	34	
51-70 ans	16	66,7	8	33,3	24	
Total	60	68,2	28	31,8	88	
**Statut professionnel**						**0,759**
Actifs	54	67,5	26	32,5	80	
Inactifs	6	75,0	2	25,0	8	
Total	60	68,2	28	31,8	88	
**Durée en hémodialyse**						**0,008**
< 1 an	8	33,3	16	66,7	24	
1-4 ans	36	78,3	10	21,7	46	
>= 5 ans	16	88,9	2	11,1	18	
Total	60	68,2	28	31,8	88	
**Paramètres de comorbidités**						**0,221**
Hypertension artérielle	32	80,0	8	20,0	40	
Diabète	10	71,4	4	28,6	14	
Cardiopathie	6	37,5	10	62,5	16	
Ulcère gastroduodénal	8	80,0	2	20,0	10	
Pas de comorbidité	4	50,0	4	50,0	8	

## Discussion

### Caractéristiques généraux

Notre étude a montré que la population hémodialysée était jeune au Togo. L’âge moyen de nos patients (38,8 ans) est similaire à ceux retrouvés en Afrique noir. En effet, SABI et al. [6] avaient trouvé dans leur étude une moyenne d’âge de 49,5 ans. Au Sénégal en 2015, NDIAYE et al. [7] ont retrouvé une moyenne d’âge de 44,73 ans; au Burkina Faso, LENGANI et al. ont trouvé une moyenne d’âge de 40 ans [8]. Des travaux réalisés dans les pays occidentaux ont montré que les patients en hémodialyse chronique étaient plus âgés, au-delà de 60 ans [9, 10]. Cette différence peut être liée à l’avancée médicale des pays développés, entrainant une espérance de vie plus longue avec des pyramides des âges plus larges au sommet. Elle pourrait également être due à la non maitrise des facteurs de risque cardiovasculaires, pourvoyeurs d’insuffisance rénale terminale dans nos pays en développement. La majorité des patients (90,9%) étaient des travailleurs. NDIAYE et al [7] au Sénégal avaient retrouvé 68,7% de patients inactifs. Cette différence pourrait résulter d’un biais dans notre sélection de mise en dialyse. En effet les séances de dialyse étant onéreuses (34 000 FCFA / séance de dialyse soit 52,44€), très proche du salaire minimum interprofessionnel garanti (SMIG= 38 000 FCFA soit 58€), seuls les patients travailleurs arrivaient à être observants. Elle pourrait aussi s’expliquer par l’inégalité de taille de l’échantillon. Nous avons retrouvé une prédominance masculine avec un sex ratio de 1,6. SABI et al [6] au Togo, DIALLO et al [11] au Mali ont trouvé des résultats similaires à savoir respectivement un sex-ratio de 1,82 et 2. Des résultats contraires existent en Afrique. En Tunisie, MOUHAMED et al [12] avaient retrouvé une prédominance féminine (sex-ratio de 0,97) en 2004. Dans l’étude de NDIAYE et al. [7] au Sénégal, il y avait aussi une prédominance féminine (sex-ratio de 0,886).

### Comorbidités

Dans notre étude, près de la moitié des patients (45,4%) avaient une Hypertension artérielle. Ce résultat est comparable à celui de NDIAYE et al. [7] au Sénégal qui avaient trouvé 68,9% de patients hypertendus dans leur série. SABI et al. [13] avaient déjà trouvé que l’hypertension artérielle était la première cause de l’insuffisance rénale chronique au Togo. Soixante-huit virgule deux pour cent des patients de notre échantillon ont présenté une dépression. Notre résultat est comparable aux données de la littérature qui rapportent une prévalence de 20 à 67% de dépression chez les hémodialysés [7, 14, 15]. Parmi les patients déprimés, 47,7% avaient une dépression sévère; 15,9% une dépression modérée et 4,6% une dépression légère. Aucun de ces patients n’a bénéficié d’un traitement antidépresseur. Ceci s’explique d’une part par le manque de recherche systématique des symptômes de la dépression chez les hémodialysés chroniques dans notre contexte où il n’existe que quatre psychiatres dans tout le pays; et d’autre part les patients hémodialysés répondent qu’ils ne sont pas « fous » à toute suggestion de consultation psychiatrique. La psychiatrie reste encore stigmatisante dans nos milieux. La crainte d’être l’objet de pitié ou de frayeur et le regard des autres qui actualisent souvent leurs différences, incitent beaucoup d’entres eux à appauvrir progressivement leurs relations sociales [16]. La survenue de la dépression était significativement liée à la longue durée de l’hémodialyse (p= 0,008). Cela s’explique par le fait que l’hémodialyse chronique est chronophage et budgétivore alors que le pouvoir d’achat du togolais est faible avec un SMIG de 35000 FCFA (52,44€). La dépression dans notre série était plus retrouvée chez les hommes que chez les femmes; ce qui est contraire aux données de la littérature [7, 11]. En effet pour ces auteurs [7, 11] la dépression était plus retrouvée chez la femme à cause de leur plus grande sensibilité et au manque d’engagement professionnel. Dans notre série malgré que la majorité des patients étaient actifs, la survenue de la dépression n’était pas statistiquement significative (p=0,759). La durée en hémodialyse reste donc un facteur indéniable de survenue de la dépression.

## Conclusion

Cette étude montre que la dépression est fréquente chez les patients hémodialysés chroniques à Lomé et qu’elle est ignorée du personnel soignant. C’est un fardeau supplémentaire pour le malade qui subit déjà le coût et la durée de l’hémodialyse chronique. La prise en charge du patient hémodialysé doit être multidisciplinaire. La collaboration entre psychiatres et Néphrologues doit débuter le plus tôt possible même avant le début de la mise en dialyse afin d’informer le patient sur les difficultés qui risquent de surgir au cours de la prise en charge.

### Etat des connaissances actuelle sur le sujet

- La dépression est fréquente chez les hémodialysés;
- La dépression est plus retrouvée chez les femmes;
- La prise en charge est multidisciplinaire.

### Contribution de notre étude à la connaissance

- La prévalence de la dépression au Togo (première étude réalisée dans l’unique centre publique du pays);
- La survenue de la dépression était liée à la durée en hémodialyse;
- La dépression était plus retrouvée chez les hommes que chez les femmes.
